# Single Center Analysis of Japanese Homecoming Delivery and Postpartum Depression

**DOI:** 10.31662/jmaj.2022-0069

**Published:** 2022-05-30

**Authors:** Shunji Suzuki

**Affiliations:** 1 Department of Obstetrics and Gynecology, Japanese Red Cross Katsushika Maternity Hospital, Tokyo, Japan

**Keywords:** grandparents, partner, homecoming delivery, postpartum depression, Japan

Childrearing support from grandparents has been reported to be associated with positive infant attachment ^(1)^. For example, a grandmother will provide breastfeeding advise and potential influence on decision-making regarding childrearing to her daughter (= new mother).

In Japan, the formation of nuclear families in cities has been rapidly becoming commonplace. They live only with their partner and cannot receive childrearing support from their grandparents; however, in Japan there has been a tradition of homecoming delivery called “satogaeri bunben” ^(1), (2)^. The definition and characteristics of the Japanese homecoming delivery were defined as follows: a pregnant woman returns to her family home for the delivery at 32-34 weeks of gestation and stays with her parents and family members to get sufficient support and to rest physically and psychologically until a couple of months after delivery. Experience, skill, and wisdom about care of the baby can be passed on from the grandmother to a new mother. The significance of the tradition for the new mother in the puerperal period has been considered to promote acquisition and learning of parenting techniques and reduce anxiety about childrearing ^(3)^.

Although several studies had investigated the positive effect of grandparents’ support of their daughters on the grandchildren, some disadvantages in the traditional delivery have been suggested ^(2), (3)^ because the new mother has to live separately from her husband until she returns to her marital house with the newborn baby ^(2)^.

In this study, we examined the influence of the presence of grandparents and/or partner, and/or the tradition on the maternal mental status at 1 month after delivery.

The study protocol was approved by the Ethics Committee of the Japanese Red Cross Katsushika Maternity Hospital (K2019-28). Patients’ informed consent for the publication was obtained.

We reviewed the obstetric records of all nulliparous healthy women with vaginal singleton delivery at term at our institute between January and June 2019 (n = 356, Figure 1). We excluded cases of multiparous women, multiple births, Cesarean deliveries, mothers with history of mental disorders and/or pregnancy depression, mothers with low-birth-weight infants, and mothers whose babies were admitted to the neonatal intensive care unit because they have been already reported to be associated with postpartum depression ^(4), (5), (6)^. The 356 subjects were divided into 3 groups based on the contents of the questionnaire at their first visits: (1) those living near our institute with the presence of grandparents living together or nearby (group A, n = 200, 56%), (2) those living near our institute without the presence of grandparents living nearby (group B, N = 53, 15%), and (3) those with homecoming delivery (group C, n = 103, 29%).

**Figure 1.**
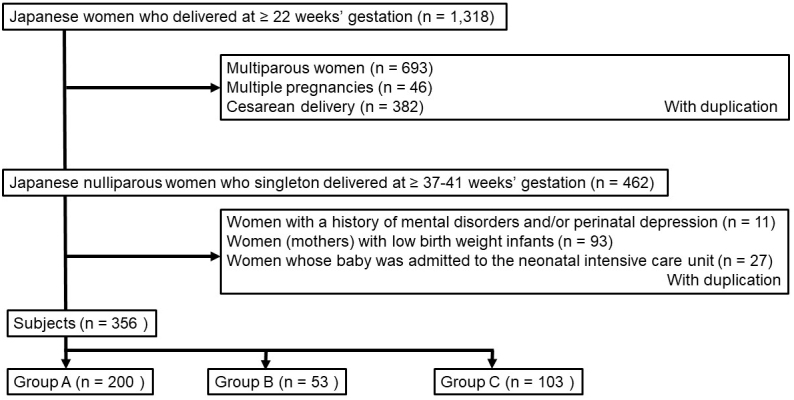
Flow diagram of the study.

The maternal mental status was evaluated based on the scores of the questionnaires of the Edinburgh Postnatal Depression Scale (EPDS). The EPDS was self-completed in an own space of every woman based on the feelings during the previous 7 days. In this study, women with EPDS scores of 9 points or more were regarded as “positive screening” according to the results of previous observations in Japan ^(7)^.

Data are expressed as mean ± SD or number (%). For the statistical analysis, the *Χ^2^* test for categorical variables and one-way analysis of variance for continuous variables were employed. The SPSS Statistics software version 20 (IBM Corp., Armonk, NY, USA) was used for the statistical analyses. Differences with *P* values <0.05 were considered significant.

The average maternal age and EPDS scores and the incidence of the EPDS scores of 9 points or more at 1 month after delivery in the 3 groups are presented in Table 1. No significant differences were observed in these valuables among the three groups.

**Table 1.** Maternal Age, Average EPDS Scores, and Incidence of the EPDS Scores of 9 Points or More at 1 Month after Singleton Healthy Delivery in the Nulliparous Healthy Women.

	Group A	Group B	Group C	Total
Number	200 (56)	53 (15)	103 (29)	356 (100)
Maternal age (y)	32.5 ± 6.4	33.0 ± 5.9	32.2 ± 6.4	32.5 ± 6.6
Average EPDS score	4.3 ± 3.4	4.3 ± 3.6	4.4 ± 3.7	4.3 ± 3.6
EPDS score ≥ 9	30 (15.0)	7 (13.2)	16 (15.5)	53 (14.9)

Data are expressed as number (percentage) or average ± standard deviation. Group A: Women living near our institute with the presence of grandparents living together or nearby. Group B: Women living near our institute without the presence of grandparents living nearby. Group C: Women with homecoming delivery.

The incidence of the EPDS scores of 9 or more at 1 month after vaginal delivery was 14.9%. This prevalence may be higher than that in some previous reports in Japan ^(2), (3), (7)^; however, it seemed to be consistent with that in a recent Japanese repor ^(8)^. It is important to keep in mind that the EPDS results will be influenced by a variety of environmental factors, including maternal characteristics, perinatal/obstetric outcomes, and regional differences ^(8), (9)^. We cannot deny the possibility that the reason for the current prevalence of women with depressive symptom is an underestimation of the relationship with the target explanatory variables. However, above all, we should understand that the EPDS is not intended to provide a diagnosis ^(7)^.

In this study, no significant differences were observed in postpartum depressive status regardless of the presence of grandparents or the place of delivery. Otherwise, the absence of grandparents or partners may not be a major factor associated with the postpartum mental status of new mothers at 1 month after delivery. It was a surprising impression for us because grandparents who can quickly notice the problems of childrearing and the thoughts of new mothers have been thought to be as thankful presence. Otherwise, in Japan, the new mothers might have selected birthing place that suits their mental status considering the presence or absence of their grandparents or partners. Furthermore, the mental burden in the pregnant women based on the current living environment in Japan may not be solved by the presence of grandparents or partners alone.

We understand that this study has several limitations other than a small number of subjects based on some previous reports on the incidence of postpartum depression, although the sample size was calculated to be at least sufficient for statistical calculations ^(8), (9)^. First, the current examination was limited to 1 month after delivery. Mothers with homecoming delivery will return to the place of residence where their partners live around the second month of their deliveries. New mothers sometimes depend on their own family members, which makes them feel anxious about childrearing when and after they have to leave their original families ^(2)^. The period of 3-4 months after delivery has been reported to be the time when the maternal mental status most deteriorates during the first year of postpartum ^(10)^. At the point, they will return to their former routine livings from the situation protected by their parents. Therefore, different results may have been obtained if the same examination was conducted at 2-4 months after delivery. Although homecoming delivery is a good support system, it may have some disadvantages. To date, home delivery has been suggested to provide support for healthy mothers. In a recent article by Takahashi Y et al. ^(11)^, homecoming delivery seemed to be associated with the low incidence of maternity blues. Maternity blues is a self-limited condition that shortly begins after delivery and can present with a variety of symptoms, such as tearfulness ^(12)^. If symptoms affect daily functioning or last longer than 2 weeks, there is a possibility of developing postpartum depression. In this study, we did not investigate the maternal mental status at 4-5 days after delivery; however, the mental health support gained from homecoming delivery may be temporary. This may also be supported by the same tendency in the EPDS scores observed in their study ^(11)^. In addition, we understand the presence of other limitations concerning the characteristics of subjects that may affect the mothers’ mental health such as occupation, the presence of friends or other people to consult, and the sex of their children. These are the items that should be asked to support the mothers with depressive symptoms ^(4), (5), (6), (8), (9)^; however, unfortunately, we did not dare to ask the women who have parents or partners about them.

In recent years in Japan, it cannot be denied that there has been a large intergenerational disparity in values between mothers and daughters ^(13), (14)^. As the age of first marriage increases, the number of cases in which parents have already died or are not in a state where they can support childrearing may also possibly increase ^(2)^. In addition, many new mothers have been reported to feel disagreement with their grandparents about childrearing, such as the presence of excessive interference ^(15)^. There may be no differences on average in the mental status among the three groups in this study under the emotional variations in individual families.

Finally, in Japan, there is the Postpartum-Care Project, a system that subsidizes childrearing support with public expense by the Japanese Ministry of Health, Labour and Welfare (https://www.mhlw.go.jp/index.html). However, since the project is carried out on a municipal basis, in principle, it does not subsidize the mothers without resident’s card with homecoming delivery from other areas. Based on the current result, no significant differences were observed in postpartum depressive status regardless of homecoming delivery. Therefore, the target of the subsidies for the postpartum care should be all new mothers, not limited to those with resident’s card of the municipality.

In conclusion, there seemed to be no significant differences in the incidence of postpartum depressive status regardless of the presence of grandparents and partner or the place of delivery in Japan.

## Article Information

### Conflicts of Interest

None

### Author Contributions

Shunji Suzuki: project development, data management, data analysis, and manuscript writing/editing.

### Informed Consent

The study protocol was approved by the Ethics Committee of the Japanese Red Cross Katsushika Maternity Hospital (K2019-28).

Patients’ informed consent for the publication of this report was obtained.
